# Molecular Analysis of Serum and Bronchoalveolar Lavage in a Mouse Model of Influenza Reveals Markers of Disease Severity That Can Be Clinically Useful in Humans

**DOI:** 10.1371/journal.pone.0086912

**Published:** 2014-02-05

**Authors:** Yadunanda Kumar, Cui Liang, Gino V. Limmon, Li Liang, Bevin P. Engelward, Eng Eong Ooi, Jianzhu Chen, Steven R. Tannenbaum

**Affiliations:** 1 Interdisciplinary research group in Infectious disease, Singapore-MIT Alliance for research and technology (SMART), Singapore, Singapore; 2 Department of Biological Engineering, Massachusetts Institute of Technology, Cambridge, Massachusetts, United States of America; 3 DUKE-NUS Graduate Medical School, Singapore, Singapore; 4 Koch Institute for Integrative Cancer Research and Department of Biology, Massachusetts Institute of Technology, Cambridge, Massachusetts, United States of America; University of Tübingen, Germany

## Abstract

**Background:**

Management of influenza, a major contributor to the worldwide disease burden, is complicated by lack of reliable methods for early identification of susceptible individuals. Identification of molecular markers that can augment existing diagnostic tools for prediction of severity can be expected to greatly improve disease management capabilities.

**Methodology/Principal Findings:**

We have analyzed cytokines, proteome flux and protein adducts in bronchoalveolar lavage (BAL) and sera from mice infected with influenza A virus (PR8 strain) using a previously established non-lethal model of influenza infection. Through detailed cytokine and protein adduct measurements of murine BAL, we first established the temporal profile of innate and adaptive responses as well as macrophage and neutrophil activities in response to influenza infection. A similar analysis was also performed with sera from a longitudinal cohort of influenza patients. We then used an iTRAQ-based, comparative serum proteome analysis to catalog the proteome flux in the murine BAL during the stages correlating with “peak viremia,” “inflammatory damage,” as well as the “recovery phase.” In addition to activation of acute phase responses, a distinct class of lung proteins including surfactant proteins was found to be depleted from the BAL coincident with their “appearance” in the serum, presumably due to leakage of the protein following loss of the integrity of the lung/epithelial barrier. Serum levels of at least two of these proteins were elevated in influenza patients during the febrile phase of infection compared to healthy controls or to the same patients at convalescence.

**Conclusions/Significance:**

The findings from this study provide a molecular description of disease progression in a mouse model of influenza and demonstrate its potential for translation into a novel class of markers for measurement of acute lung injury and improved case management.

## Introduction

Influenza virus infections result in a contagious respiratory illness with highly varied outcomes among the human populations. While a significant proportion of the populations worldwide recover from influenza within 2 weeks, susceptible individuals can have a variety of severe and potentially lethal complications including pneumonia, bronchitis and acute respiratory distress syndrome (ARDS). Despite the development of vaccines and therapeutics, influenza epidemics continue to pose a major challenge to worldwide disease management efforts due to periodic emergence of drug-resistant influenza strains or new strains to which pre-existing immunity is low or absent. One example is the pandemic influenza H1N1 swine flu strain which is circulating worldwide since its emergence in 2009 and causes illnesses similar to the seasonal H3N2 influenza strains [1].

The most common symptoms of influenza include fever, runny nose, sore throat, cough, lethargy but occasionally more severe respiratory complications are reported which could be lethal without early detection and treatment. While some of the risk factors that trigger severe influenza have been identified as age, with individuals at the extreme of age at higher risk, other underlying co-morbidities such as obesity, heart disease also elevate the risk of poor clinical outcomes [2]. Although the mechanisms that trigger these complications of influenza are poorly understood a combination of viral virulence as well uncontrolled inflammatory responses to the virus has broadly been suggested to be a predominant mechanism for severe lung pathology from influenza [3]. A number of studies have attempted to unravel the mechanisms contributing to lethality using mouse models of influenza which replicate many pathological features of human pandemics including acute immune responses, inflammatory lung damage and potentially fatal pneumonia [4], [5]. Recent reports have employed systems biology approaches to indicate that lethality in mice may be primarily driven by a self-amplifying inflammatory neutrophil response triggered by the virus [6]. Similar studies in more relevant human cohorts are significantly limited both by lack of accurate methods to measure acute lung injury and the large variations in individual response to the viral infections. Several studies have examined correlation of disease parameters such as viral shedding and symptom scores with levels of IL-6 [7], [8], an acute phase cytokine which was recently reported as a potential biomarker of severity in pandemic influenza infections [9]. While providing important insights into potential determinants of pathology in humans, these markers have limited specificity and are unable to inform on specific lung injuries relevant to influenza infection in a clinical setting. Notwithstanding the diverse factors that result in severe outcomes in influenza patients, it is generally recognized that management of disease severity in influenza is greatly improved by early detection and treatment.

Development of biomarkers that can model one or more features of disease severity can additionally be expected to facilitate early prediction and therefore treatment. In this report, we have attempted to bridge the gap between mouse and human influenza infections. Through comprehensive molecular analysis of sera and bronchoalveolar lavage (BAL) from a mouse model of influenza, as well as a longitudinal cohort of influenza patients, we have identified molecular markers of disease progression of influenza that may translate to clinically useful markers of infection-induced lung injury in humans.

## Methods

### The mouse model of influenza infection

C57BL/6 (B6) mice at 8–12 weeks of age were purchased from the Centre for Animal Resources (CARE), Singapore. Influenza A/Puerto Rico/8/34 H1N1 virus (PR8) were provided by Professor Vincent TK Chow (National University of Singapore, Singapore). Mice were infected with a sub-lethal dose of influenza virus by intra-tracheal administration after anesthetization. At indicated time points mice were euthanized and sera were collected. BAL was performed using 1 ml sterilized PBS for 3 times per mouse, the collected fluids were pooled. The BAL fluids were centrifuged at 5,000×g for 10 min and the supernatants were frozen at −80 deg.

This study was carried out in strict accordance with the National Advisory Committee for Laboratory Animal Research (NACLAR) Guidelines (Guidelines on the Care and Use of Animals for Scientific Purposes) in facilities licensed by the Agri-Food and Veterinary Authority of Singapore (AVA), the regulatory body of the Singapore Animals and Birds Act. The protocol was approved by the Institutional Animal Care and Use Committee (IACUC), National University of Singapore. Mice were monitored every day after injury. At different time points, a group of mice were euthanatized by injection of ketamine/meditomidine, the lung samples were then collected for experiments. Any mouse with 30% body weight loss will be euthanatized immediately.

### Influenza patient recruitment, sample collection and clinical evaluation

The early dengue infection and outcome (EDEN) study is a multi-center longitudinal study of adult febrile infections that was carried out at a number of clinics island-wide in Singapore [10]. As the main inclusion criteria in this study was fever, over 20% of the patients enrolled were eventually found to be negative for dengue but positive for influenza A virus.Enrollment of eligible individuals was based on written informed consents and the protocols were approved by the National Healthcare Group (DSRB B/05/013). The study protocols for the study have been described earlier [10]. In brief, adult patients (>21 years) presenting with acute onset fever (≥38.0°C for less than 72 hours) without rhinitis were included in the study. NP swabs collected were tested for viral pathogens by direct immunofluorescence assay (DFA) and virus isolation using R-Mix^TM^ cells in shell vials (Diagnostic Hybrids Inc., Athens, Ohio) according to the manufacturer's instructions. The D^3^ Ultra DFA Respiratory Virus Screening and ID Kit (Diagnostic Hybrids Inc., Athens, Ohio) to detect for influenza A and B, RSV, human metapneumovirus, adenovirus, and parainfluenza 1, 2 and 3 in both DFA as well as shell vial cultures.Subtyping of influenza A virus was carried out by RT-PCR usingpreviously published protocols [11], [12]. Venous blood samples were also collected at enrolment (visit-1) as well as on fever day 4 to 7 (visit-2) and weeks 3 to 4 (visit-3), aliquoted and frozen at −80°C. ‘Fever day’ here refers to number of days post onset of fever.

### Fluorescent bead-based measurement of cytokines

Cytokine measurements were performed in duplicates using either the Bioplex 23-plex mouse cytokine kit or the Bioplex 27-plex human cytokine kit from BioRad as per manufacturer's instructions. For mouse BAL fluid analysis, undiluted BAL was mixed with 20% BSA to a final concentration of 1% before use in the bioplex assay.

For human serum samples, 12.5 uL of serum was used for each measurement. The standard curves were optimized automatically by the software (Bioplex manager) and verified manually. The Bioplex manager software was used to calculate cytokine concentrations and only measurements that showed a coefficient of variability (CV) of <10% were included for further analysis. Levels of most cytokines were below detection limit in BAL obtained from Day-0 infected mice as well as in some of the late time points. For the purpose of analysis the values here were set to the lower limit of detection in each case.

### Quantitative analysis of mouse BAL proteome

#### Sample pooling, iTRAQ labeling and OFFGEL peptide separation

We performed an isobaric tagging for accurate quantitation (iTRAQ) method for multiplexed proteomic analysis of BAL fluid from infected mice. For this, BAL fluid samples from 3mice each at Days 0, 5, 14 and 21 were concentrated and the protein concentration was measured in each sample using the BCA method in a kit (Pierce chemical co. USA). Seventy-five micrograms of protein was subjected with trypsin digestion at a ratio of 1∶80 (trypsin: protein) followed by labeling with isobaric tags using the 4-plex-iTRAQ kit (AB Sciex Pte Ltd, USA) as per manufacturer's instructions. The peptides from Day-5 were labeled with 115 reporter ions while those from Days 14 and 21 were labeled with 116 and 117 reported ions. Peptides from Day-0 were labeled with 114 reporter ion which served as a reference control. The individually labeled peptide samples from each group were pooled and multiplexed peptide samples were desalted using a C-18 SPE cartridge (Agilent technologies), resolved by isoelectric focusing on a pH3–10 strip (GE healthcare) on an OFFGEL fractionator (Agilent technologies). The resolved peptides were collected in 12 fractions, dried and dissolved in 15 µl of 2%ACN/0.1%TFA.

#### LC MS/MS analysis

The samples were analyzed on an Agilent 6520 Accurate-mass QTOF-LC/MS system equipped with a 1200 series HPLC-Chip/MS system. We separated peptides on a HPLC-Chip with 75 um X 150 mm analytical column HPLC-Chip and a 160 nL enrichment column. Three injections (1 ul each with approximately 2 ug peptides) of each sample were separated using a 60 min gradient (5% at 0 min 10% 2 min, 50% 42 min 80% 42–50 min, 5% 50–60 min) with Water/0.1%formic acid as aqueous phase and 95%acetonitrile/5%water/0.1%formic acid as organic phase at flow rates of 300 nL/min. Peptides eluting from the LC were injected online into the accurate-mass QTOF and examined in positive ion mode with the following settings for MS mode: 4 spectra/sec, m/z 110–2300, MS/MS mode: 8 spectra/sec, m/z 60–1097 with drying gas flow 5 L/min, 325 degC, collision energy slope 3, intercept 2.5, and a capillary voltage of 1950.

#### Data analysis

Spectrum Mill software (Agilent Technologies) was used for protein identification and quantitation of iTRAQ reporter ion intensities. A minimum peptide score of 8 and a protein score of 10 was used to generate protein lists by searching against the Swissprot database. These thresholds were determined by comparing results from searching the Swissprot database and a reversed random database to identify error rates. The peptide and protein score thresholds indicated above ensured a false discovery rate of <5% in protein identification. Only proteins identified with two or more peptides were selected for relative quantification. A global weighted threshold for fold change was determined by comparing the ratio of summed intensities of each reporter ions for all peptides (115/114 1.13; 116/114- 1.19; 117/114- 0.91) and then the intensities of each peptide ratio were further corrected by this factor. This weighted threshold was essential to make sure the fold changes observed were not simply due to an overall bias towards one or more reporter ion. Finally, fold change for each protein was calculated as a ratio of summed intensities of reporter ion across different peptides per protein. Further, a background range for fold changes was determined by average of ratios of test samples (Days 5, 14 and 21) and control (Day 0) and was found to range between 0.7–2.1. Therefore for the final shortlist of candidates we employed a more conservative fold change range of 0.6 for down regulation and > = 3 for upregulation in relation to controls.

### Measurement of lung proteins in Mouse BAL and in patient sera

Lung proteins CC10, SPAB, SPC and SPD were detected in mouse BAL and serum samples by western blotting. Briefly, 10 ug of normalized BAL samples from Days-0, 5, 14 and 21 were separated on a 4–15% SDS-PAGE, transferred to a nitrocellulose membrane and probed with mouse-specific antibodies antibodies to CC10 (Santa Cruz, SC-9772), SPA (Santa Cruz, SC-13977), SPB (Santa Cruz, SC-133143), and SPD (Santa Cruz, SC-13980). Measurement of SPA and CC10 levels in human sera was performed by ELISA using commercially available kits specific for human SPA (Biovendor Research and Diagnostic products; SPA-RD191139200R; CC10-RD191022200)) according to manufacturer's instructions. For measurements of SPA serum samples were diluted 10 fold while for CC10 a 25 fold dilution was used.

### Measurement of serum chlorotyrosine and nitrotyrosine

Nitrotyrosine (NT) and chlorotyrosine (CT) in mouse and human serum were measured by a Liquid chromatography-triple quadrupole MS method we have previously described [13]. Briefly, 2 mg of serum protein was spiked with 4 pmol internal standards (IS) L-3-chloro-[13C9, 15N]-tyrosine and L-3-nitro-[13C9, 15N]-tyrosine, and digested in the sodium acetate solution 0.1 M (pH 7.4) with 0.4 mg pronase E (freshly treated by the size-exclusive micro bio-spin column). The mixture was incubated at 50°C for overnight (16 hrs.) and filtered by Vivospin500 3KMW centrifuge filter at 15,000 rpm to remove undigested protein. The amino acids were further purified by Agilent 1200 series HPLC system (Waldbronn, Germany) on an Xbridge TM Phenyl column (3.5 µm, 4.6×50 mm, Waters, Milford, MA). The fractions containing nitrotyrosine and chlorotyrosine, together with internal standards, were collected and dried by SpeedVac for subsequent LC/MS/MS analysis. Subsequent mass spectrometry analysis of target compounds involved separation on an Xbridge TM Phenyl column (3.5 µm, 1.0×100 mm, Waters, Milford, MA) online injection into an Agilent 6460 triple quadrupole mass spectrometer. Two microliters of each sample was injected and eluted by isocratic 25% methanol (0.1% formic acid) for 13 min at 15 µL/min. CT along with IS were analyzed by regular multiple reaction monitoring (MRM) as follows: 216/170 (CT) and 226/179 (CT, IS). NT along with IS were measured by modified MS3 based in-source fragmentation as follows: 181/117 (NT) and 190/125 (NT-IS) by elevating the potential to 135V at the ion source. The limits of quantitation achieved were 8.1 and 7.3 nM for CT and NT, respectively.

### Statistical analysis

K-means clustering was performed on time courses of measurements using the Unscrambler-X statistical software package (CAMO software, Oslo, Norway). In order to perform K-means clustering of time courses of cytokines levels over different time points, cytokines levels were first normalized across the three time points by dividing the values by the means across the time points. It was empirically found that K = 4 gives the best inter-class variances. In a second step, the members across different clusters were compared manually to determine if the fold changes of cytokines levels between visit 1 and 2, and visit 2 and 3 were statistically significant (p<0.05); if not, the clusters were merged. This resulted in three broad clusters as shown in the results section. The Willcoxon rank sum test was used for estimating the significance of differences between population means. In general p<0.05 was considered significant. Correlation analysis was performed using the Pearson's correlation matrix on the Graphpad prism software and where indicated, the correlation coefficient and the significance values are reported. ROC curves were generated using Graphpad prism software.

## Results

### I. Cytokines and protein adducts identify distinct disease states in a mouse influenza model

**Figure 1:**
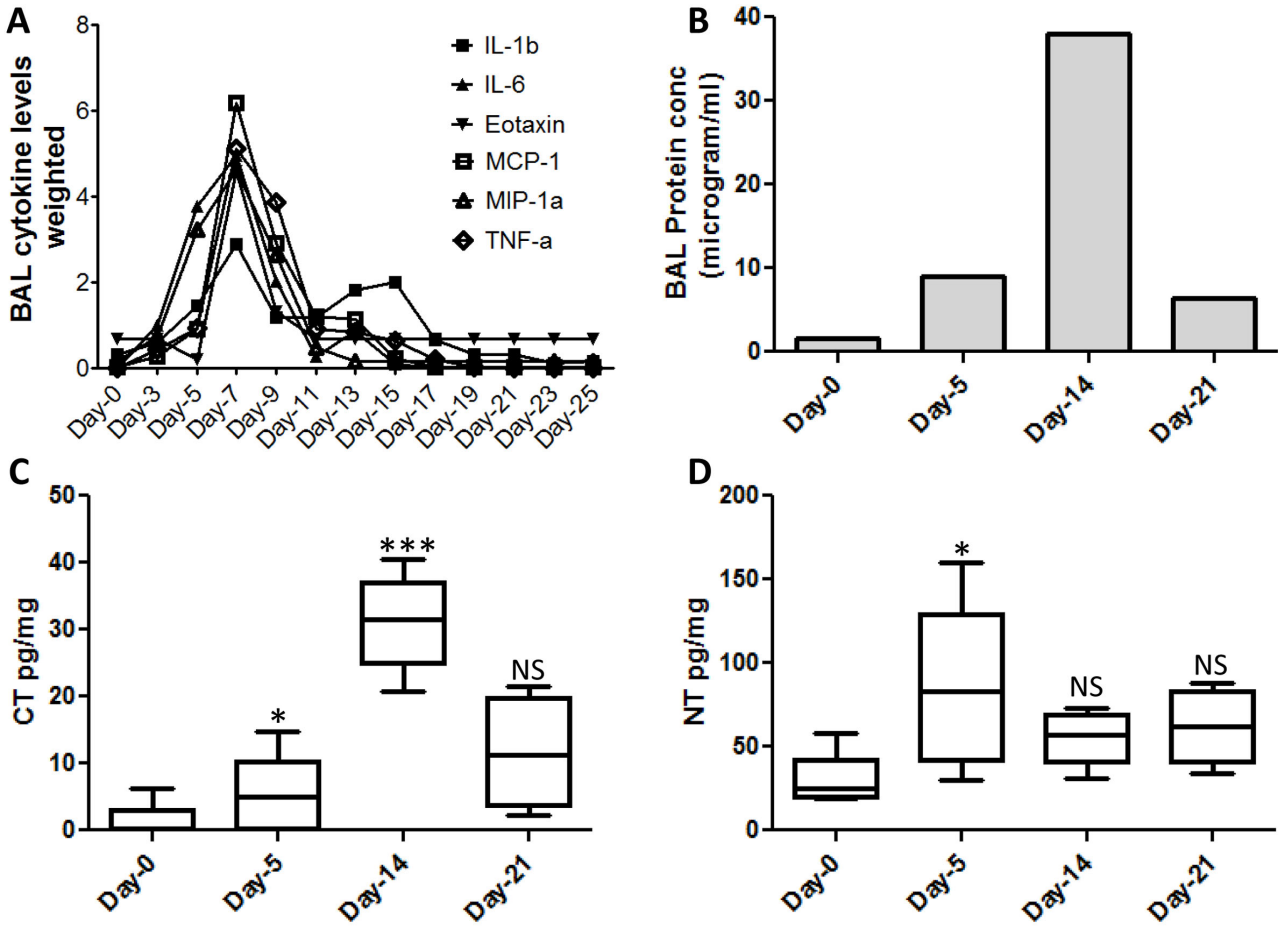
Stages of disease progression in mice infected with influenza virus. A. Changes in inflammatory BAL cytokines and chemokines measured at 2-day interval till day 22 post infection. The Y-axis shows cytokine levels that have been normalized by dividing with mean. B. total protein content of the BAL during influenza infection in mice. C. Levels of CT in mouse serum displayed as pg per milligram of protein. D. Levels of NT in mouse serum displayed as pg per milligram of protein. Statistical confidence measure using Mann Whitney test with p values 0.05 (*), 0.005 (***) indicated on the box plot.

A detailed measurement of levels of 23 cytokines in the BAL, at 2-day intervals between days-0 to 25 revealed maximal elevation of inflammatory cytokines IL1a, IL1b, IL6, TNFα, IFNγas well as chemokines mKC, Eotaxin, MIP1a, MIP1b and MCP1 during the days 5–9 of infection (Fig-1A & Fig-S1). BAL protein concentration has been extensively used as an indirect though crude measure of lung inflammation [14]. In our mouse model, the total BAL protein concentration increased significantly to peak at day-14 and by day-21 the levels decreased by 5-fold, consistent with maximal lung damage and recovery during these stages respectively (Fig 1-B). Protein adducts formed from nitric oxide chemistry have emerged as sensitive indicators of neutrophil and macrophage activity [15]. We measured levels of two protein adducts 3-chlorotyrosine (CT) and 3-nitrotyrosine (NT), in sera from infected mice at days-0, 5, 14 and 21, using a mass spectrometry-based method. Compared to controls, serum NT levels were maximally elevated at day-5 (Fig-1C). In contrast, levels of serum CT were increased significantly between days 5 and 14 and appeared to rapidly decrease at day-21 (Fig-1D). The overall accumulation of CT adducts at day-14 is consistent with maximal neutrophil infiltration during this stage (data not shown). Together, the above data as well as previously published information on this model [16] established the kinetics of viral replication and clearance, inflammatory lung damage as well as recovery in our non-lethal mouse model of infection with influenza virus PR8 strain.

### II. Proteomic analysis of mouse BAL during influenza infection

**Figure 2:**
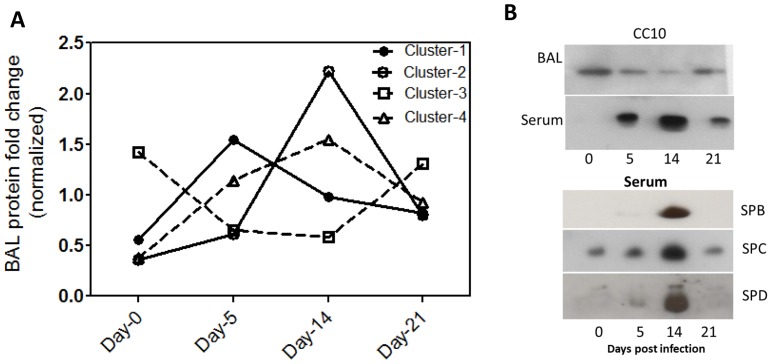
Dynamics of BAL proteome at different stages of influenza infection. A. K-means cluster analysis of proteins that exhibited significant fold change across Days 5, 14 and 21 when compared with Day-0 of influenza infection. The Y-axis indicates a weighted value that is the individual value divided by mean across the four time stages. B. Immunoblot analysis of mouse BAL and serum to evaluate levels of selected lung proteins that were depleted from BAL during peak viraemia and inflammation.

We performed an iTRAQ based quantitative proteomic analysis of BAL from mice infected with influenza for different lengths of time. In particular, we evaluated stages we have shown to correlate with peak viremia, inflammatory lung damage and recovery of lung function both in this study, and in previous reports [16], [17]. From the iTRAQ analysis we identified a total of 113 proteins, of which 42 satisfied our filtering criteria of proteins that exhibited fold changes > = 3 or < = 0.6, compared with control, were identified with 2 or more peptides, and were not immunoglobulin components (Table-1). A K-means clustering analysis identified overall four distinct clusters whose levels changed differently across different stages of disease (Fig-2A). Cluster-1 consisted of 5 proteins that were maximally elevated at day-5 and remained elevated at day-14, returning to near control levels by day-21. These included a majority of acute phase reactants and complement proteins. Cluster-2 consisted of 7 proteins that were maximally elevated during day-14, a stage coinciding with peak neutrophil infiltration and lung damage. These consisted of several iron-binding proteins, complement proteins, and hemoglobin isoforms indicative of a significant increase in vascular permeability resulting in global influx of serum proteins. Cluster-4 consisted of 21 proteins that were elevated to a lesser extent in Day-5 and remained elevated through all stages. A separate cluster of proteins, Cluster-3, was observed consisting of 9 proteins that were progressively depleted from days-5 and 14 with some returning to control levels by day-21. Interestingly this cluster consisted of mostly resident BAL protein such as uteroglobin/CC10, Surfactants SPA, SPB, SPD, glutathione S-transferase isoform omega-1, lysozyme C2. It is noteworthy that among proteins identified by single peptide hits (Supplementary Table S1), several proteins of analogous function to the above, such as glutathione S-transferase isoform-1, Cu-Zn superoxide dismutase, were also depleted indicating that possibly a broader range of resident BAL proteins are depleted during acute and inflammatory stages of influenza infection. Immunoblot analysis of CC10 confirmed its reversible depletion from BAL during days-5 and 14 (Fig-2B). Interestingly, when we examined in parallel sera from the same mice we found an inverse profile wherein the CC10 that is normally absent or undetectable in sera, reversibly increased to a detectable level at day-5 and day-14 (Fig-2B). A similar profile in serum was observed for several surfactant proteins as seen by immunoblots (Fig-2B). Overall, a significant number of BAL proteins were identified whose levels changed dramatically around the time during the time of maximal lung inflammation.

**Table 1:** BAL proteome flux in a non-lethal mouse model of influenza infection.

BAL Protein	Swissprot	No.of	Fold Change over Day-0			Cluster
	Accession	peptides	Day-5	Day-14	Day-21	Group*
Serum albumin	P07724	31	5.32	6.79	4.41	4
Serotransferrin	Q921I1	28	7.22	11.41	8.48	4
Complement C3	P01027	28	3.83	3.07	1.65	4
Hemopexin	Q91X72	16	3.34	5.54	2.16	4
Alpha-2-macroglobulin	Q61838	14	5.72	5.34	2.45	4
Ceruloplasmin	Q61147	14	2.65	4.45	2.45	4
Vitamin D-binding protein	P21614	12	4.34	5.12	2.48	4
Apolipoprotein A-IV	P06728	10	2.79	4.47	2.58	4
Liver carboxylesterase N	P23953	9	2.18	3.16	1.94	4
Fibronectin	P11276	9	2.41	6.95	4.77	2
Apolipoprotein A-I	Q00623	8	4.03	4.57	2.47	4
Haptoglobin	Q61646	8	26.86	4.24	9.09	1
Kininogen-1	O08677	7	3.2	4.85	2.8	4
Antithrombin-III	P32261	7	1.93	3.21	2.21	4
Hemoglobin subunit alpha	P01942	5	0.81	4.93	1.14	2
Beta-2-glycoprotein 1	Q01339	6	3.77	6.13	3.07	4
Chitinase-3-like protein 3	Q35744	6	0.48	0.49	1.45	3
Murinoglobulin-1	P28665	5	4.3	4.9	2.46	4
Gelsolin	P13020	5	2.03	14.11	4.47	2
Actin, cytoplasmic 2	P63260	6	3.67	3.15	1.36	4
Alpha-2-HS-glycoprotein	P29699	5	2.25	3.07	1.87	4
Hemoglobin subunit beta-1	P02088	4	0.92	5.07	1.74	2
Clusterin	Q06890	4	3.55	3.55	2.29	4
Plasminogen	P20918	4	2.97	3.45	3.1	4
Pulmonary surfactant-associated protein D	P50404	4	0.6	0.55	1.2	3
Uteroglobin	Q06318	3	0.2	0.13	0.4	3
Chitinase-3-like protein 1	Q61362	3	22.03	11.23	5.2	1
Pulmonary surfactant-associated protein B	P50405	3	0.56	0.3	0.49	3
Afamin	Q89020	3	2.56	4.43	2.13	4
Fibrinogen beta chain	Q8K0E8	3	5.87	4.6	1.68	1
Complement factor B	P04186	3	3.52	3.54	2.25	4
Glutathione S-transferase omega-1	P78417	3	0.23	0.27	0.91	3
Apolipoprotein E	P08226	3	1.78	6.24	3.45	2
Lysozyme C-2	P08905	3	0.53	0.41	0.77	3
Complement C4-B	P01029	3	3.6	2.63	1.63	1
Selenium-binding protein 2	Q63836	3	0.54	0.57	0.76	3
Complement factor H	P06909	3	4.33	9.32	1.7	2
Vomeromodulin	Q80X17	2	0.56	1.2	4.29	3
Fetuin-B	Q9QXC1	2	1.63	3.73	1.19	2
Inter-alpha-trypsin inhibitor heavy chain	Q61703	2	2.45	4.31	1.85	4
Lactotransferrin	P08071	2	3.09	1.69	2.09	1
Pulmonary surfactant-associated protein A	P35242	2	0.49	0.29	0.59	3

* Cluster grouping based on K-means clustering described in methods. # Days post infection.:

### III. Establishing kinetics of disease progression in a longitudinal cohort of influenza patients

**Figure 3:**
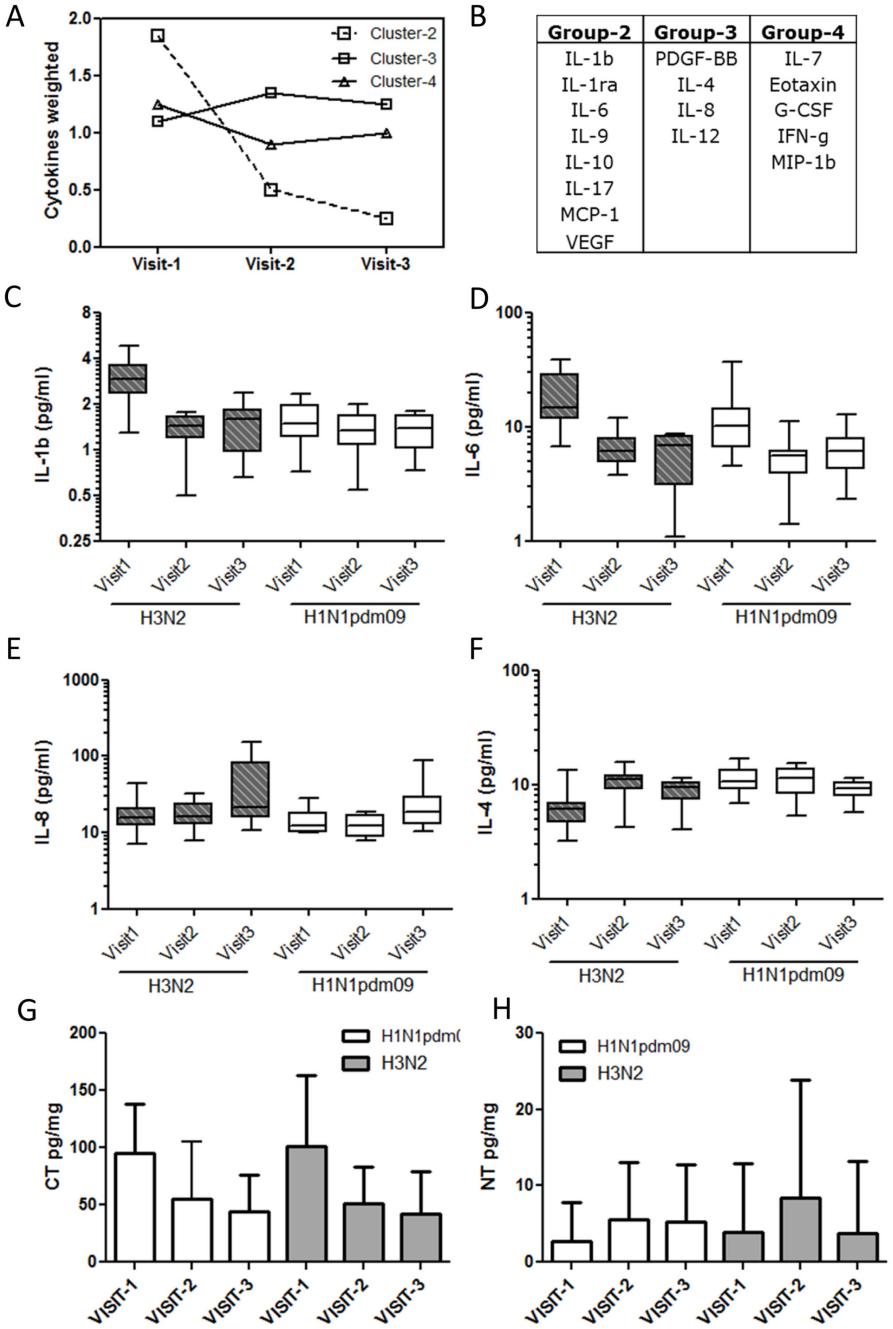
Molecular tracking of disease progression in a human cohort of influenza patients. K-means cluster analysis of cytokines measured in sera of influenza patients at three stages of infection as indicated. Average profile of changes in each luster (A) as well as list of cytokines in each cluster as indicated. Changes in a selected list of cytokines including IL1b (C), IL6 (D), IL-8 (E), IL4 (F). Levels are indicated as picogram per ml of serum. Levels of protein adducts 3-chlorotyrosine (CT) (G), and 3-nitrotyrosine (H) shown as picogram per milligram protein. Statistical confidence measure using Mann Whitney test with p values 0.05 (*), 0.005 (***).

Our experiments in mice indicated that influenza infection results in leakage of lung BAL proteins into the blood. Because loss of vascular permeability has been suggested to be one of the pathological features of severe influenza, we examined if the related leakage of lung surfactant proteins into serum was a more universal feature observable also in humans infected with the influenza virus. For this, we obtained serum samples from a longitudinal cohort of patients infected with two co-circulating strains of influenza virus- H1N1pdm09 and H3N2. These patients were recruited as part of the EDEN study where patients diagnosed with influenza were followed up at 48–72 hours (early febrile, Visit-1), 4–7 days and 3–4 weeks post onset of febrile symptoms and blood samples collected at these three stages from each sample [10]. The characteristics of patients selected for our study are indicated in Table-2. Although the longitudinal nature of this cohort allowed us to evaluate disease progression analogous to that in mice, direct comparison of disease progression in mouse and human models is complicated by several factors including- 1) qualitatively and quantitatively different immune responses to the virus, 2) different viral loads as well as replication kinetics. Therefore as a first step we performed a detailed evaluation of cytokines and protein adducts in our patient population to establish broadly the stages indicative of inflammation and antiviral responses. We measured 23 cytokines using a multiplex kit as described in methods and performed a K-means clustering analysis across the different visits to identify common signatures (Fig-3A). In patient group infected with seasonal H3N2 strain, Cytokines and chemokines including IP-10, IL-b, IL-1ra, IL-6, IL-9, IL-10, IL-17, VEGF and MCP1 were maximally elevated during the early febrile phase returning to basal levels by visit-2 and formed one cluster. IP-10 was the most significantly elevated cytokine, increasing to >50fold at visit-1 and dropping to control levels by visit-2 (Fig-S2). A second group of cytokines including IL-7, Eotaxin, G-CSF, IFN-g and MIP-1b increased to a maximal level during visit-1 but remained at high during the subsequent visits. A third cluster of cytokines such as PDGF-b, IL-4, IL-8 and IL-12 remained at maximal level from Visits 1–3. Interestingly, a similar analysis on patient group infected with H1N1pdm09indicated that, while majority of cytokines clustered similar to the seasonal flu patients, a distinct group of cytokines including IL-1b, IL-9, IL-17 and VEGF clustered differently. A closer look at individual cytokines revealed specific differences in these as well as four other cytokines, IL-4, IL-6, IL-8, G-CSF in the two patient groups. With the exception of IL-4, all other cytokines were elevated in patients with seasonal flu compared with H1N1pdm09 during visit-1 (Fig 3-C–F). We next measured the levels of CT and NT in serum samples patients with H3N2 (n = 15) and H1N1 (n = 16) and found CT to be maximally elevated at visit-1 reducing significantly during visits 2 and 3 (Fig-3G). NT on the other hand showed highest levels during Visit-2 although the differences between the various time points were not statistically significant (Fig-3H). Overall, cytokine and protein adduct profile observed during the 48–72 hours post onset of fever (visit-1) in human patients appears analogous to the day-14 infection in our mouse model described in the previous section.

**Table 2:** Characteristics of Influenza patient study population.

Patient Groups	Virus strain	Age	Race	Blood sampling Time *
Influenza	Seasonal Flu	37±16.54	Chinese 56.2%	30±16 hours (visit-1)
(n = 26)	(H3N2)		Indian 6.2%	88±40 hours (Vist-2)
	2007–2010		Malay 25.0%	14±6 days (visit-3)
			Others 12.5%	
Influenza	H1N1 pdm09	25±9.66	Chinese 53.8%	23±22 hours (visit-1)
(n = 30)	2009–2010		Indian 15.2%	108±40 hours (Visit-2)
			Malay 15.6%	15±3 days (visit-3)
			Others 15.5%	

* Average ± SD time from fever to phlebotomy (visit-1), between visits 1 & 2 (visit-2) and between.

visits 2 & 3 (visit-3).

### IV. Lung surfactant proteins in sera of humans infected with the influenza virus

**Figure 4:**
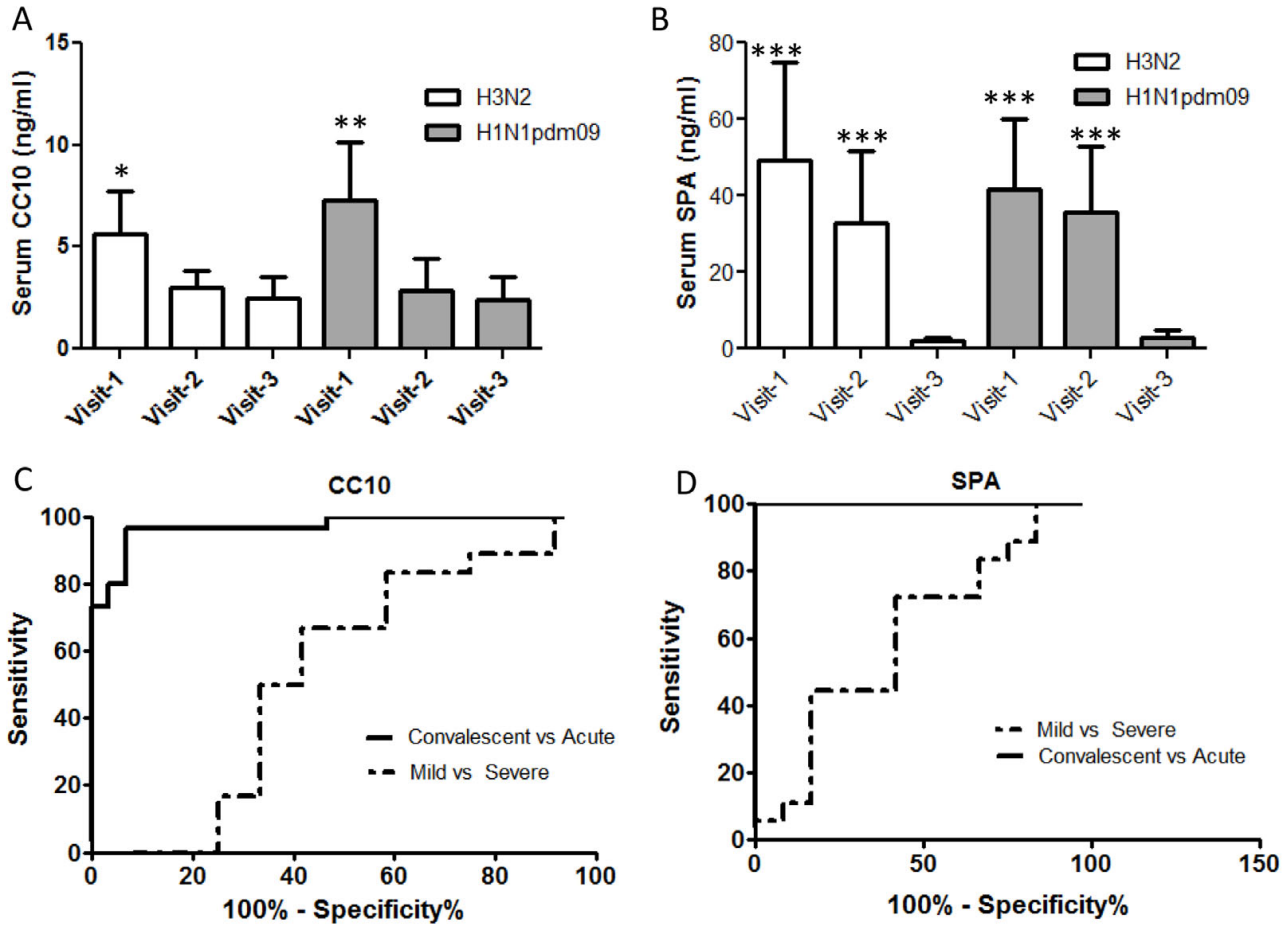
Serum levels of lung BAL proteins, CC10 and surfactant protein-A in human influenza patients. Levels of CC10 (A) and surfactant protein A (B) were measured in sera from human influenza patients using commercial ELISA kits as described in methods. The levels are indicated as ng/ml of serum and were compared with the levels measured in convalescent sera from the same group of individuals. An ROC curve analysis of each of the proteins indicated different association of these proteins with – Acute versus convalescent stages of disease (solid line) or with mild or severe disease (dashed line). Statistical confidence measure using Mann Whitney test with p values 0.05 (*), 0.005 (***).

To test the hypothesis that appearance of lung proteins in the blood during influenza may be a universal feature of the disease, we measured the levels of two BAL proteins- CC10 and surfactant protein A (SPA) in influenza patient sera using commercial ELISA kits. CC10 levels in serum were significantly higher (Fig-4A) in the influenza patients during the febrile stage (visit-1) compared with convalescence (Visit-3). A similar profile was observed for SPA which was elevated nearly 40 fold in Visit-1 compared to Visit-3 (Fig-4B). Maximal elevation of these proteins at visit-1 is consistent with the peak of inflammation during this period. Increased vascular permeability is an established feature of acute lung injury and disease severity in viral infections [18], [19]. The increase in SPA or CC10 levels in influenza patients with seasonal Flu was indistinguishable from those with H1N1pdm09 (Fig-4A–B). A receiver operator characteristics (ROC) curve analysis of SPA and CC10 in classifying patients with acute infection from convalescent states indicated excellent performance for both SPA (AUC 1.0, p<0.0001) than CC10 (AUC 0.971, p<0.05) (Fig-4C–D). We next evaluated the potential of using serum levels of lung surfactant proteins as a predictor of disease severity. Because none of the patients in our influenza cohort were hospitalized with severe illness we used duration of illness, reported by the patients, as a measure of disease severity. We classified patients with symptoms lasting 5 days or less as mild disease and those with symptoms lasting greater than 5 days as severe or prolonged illness disease. The ROC curve analysis indicated an overall poor classification for the two proteins with a marginally improved performance for SPA (AUC 0.615, p 0.29) compared with CC10 (AUC 0.537, p 0.7) (Fig-4C–D). We observed marginal (r>0.6) but highly significant (p<0.001) correlation between SPA and cytokines such as IL-6, PDBF-BB and duration of symptom.

## Discussion

The underlying mechanisms that accompany the widely varied human responses to influenza virus infections are not fully understood. We have conducted a systematic study of influenza infection centered on a well-established non-lethal mouse model to help identify early molecular markers of disease severity. The relevance of using of inbred mouse strains as hosts for experimental influenza infections has been debated and admittedly has the major disadvantage of being unable to model more complex human immune responses. Nevertheless, mouse models have been used extensively to understand mechanistic basis of pathology in influenza and provide the added advantage of removing the ‘noise’ from multiple underlying conditions and immune responses that is seen in human disease, and more suited to uncover universal features of disease pathology.

Kinetics of cytokine production, immune and inflammatory responses can vary significantly depending on viral dose, time of measurements etc [20]. Therefore, as a first step we defined stages of disease progression in our experimental mouse model by tracking the BAL cytokine flux as a measure of the early and late immune response up to 25 days post virus inoculation. Consistent with analogous reports in literature [20], [21], we observed sharp elevation of cytokines related to antiviral and innate immune pathways, as well as chemokines peaked around day-5 and correlated with drop in viral titers. Cytokine analysis of a human influenza cohort also revealed a sharp elevation of cytokines during the acute febrile phase of infection. However in the human cohort the fold increases in cytokine levels was more modest and the temporal cytokine flux was more complex. Although the significant differences in types and clinical characteristics of cohorts reported in literature prevented direct comparison of our results, elevation of inflammatory cytokines and chemokines early during presentation, and the levels we have observed, are consistent with reports in literature [22], [23], [24]. Additionally, some of these differences could also be attributed to different immune response to different viral strains. For example, levels of inflammatory cytokines IL-1b and IL-6 were greater in patients with H3N2 seasonal flu infections as compared to H1N1pdm09 strains. Nitrating oxidants play an important role in immune defense against pathogens where peroxidases such as myeloperoxidase can use reactive nitrogen species to form protein adducts such as CT and NT [15]. Neutrophils have been demonstrated to use the Myeloperoxidase-H2O2-NO2^−^ system to nitrate and chlorinate tyrosines on proteins [25]. In contrast to the short-lived reactive species, CT and NT have a longer half-life in circulation and consequently extremely useful as indicators of neutrophil activity. While both levels of both CT and NT rose significantly during day-5, CT levels continued to rise sharply to peak at Day14 suggesting that neutrophil accumulation and activity continued well past the viral clearance phase. It is unclear why NT levels at Day-14 returned to baseline, but could possibly indicate different sites of accumulation of these two adducts with NT adducts predominantly localized to lung tissue while CT is more readily observed in luminal contents such as the BAL. Interestingly, levels of CT in sera from a human influenza cohort were also much more sharply elevated than NT and peaked during the acute phase of infection. Together these results help establish a molecular description of disease progression in two very disparate systems- mouse models and a human cohort, setting the stage for detailed experiments in mouse models aimed at finding markers relevant to human disease.

We used an iTRAQ approach to quantify changes in BAL proteome during acute phase, pathological phase as well as recovery stages relative to mock-infected mice. Several previous studies have characterized the protein composition of BAL in mice and humans [14], [26] and consistent with these studies we observed that plasma proteins were the most abundant components of the BAL presumably via diffusion across the vascular barrier. We focused on identifying changes in BAL protein composition that correlate with disease progression and not surprisingly observed significant changes in more than 30 proteins correlating with acute and inflammatory phases of infection. Levels of acute phase reactants and complement proteins were significantly elevated during the acute and inflammation phases of the infection while by day-21 the levels resembled mock infected animals. Inflammation-induced loss of vascular permeability in the lungs is common in viral infections [18] and explains to a large extent the influx of plasma proteins into the BAL. Interestingly, the kinetics of protein flux we observed indicated that there was some specificity to the process with some proteins changing earlier than the others. Accumulation of hemoglobin, occurred only at day-14, later than acute phase response perhaps indicating that subtle influx of specific plasma proteins into the BAL precede a more dramatic global influx during peak inflammatory tissue damage event. The possibility that some of these proteins could be early markers of lung damage remains to be tested, although invasive nature of BAL collection procedures makes the specific use of plasma proteins in the BAL as a clinically useful marker for lung damage untenable. More interesting from a biomarker perspective was our observation that a subset of locally produced BAL proteins was depleted from the BAL during stages corresponding to the acute and inflammatory stages of disease. Several surfactant proteins including SPA, SPD and SPB as well as cc10 a lung clara cell specific protein, glutathione S-transferase and lysozyme were depleted from the BAL accompanied by their appearance in the serum in quantities detectable by western blotting.

A well-documented pathological feature of acute lung injury is the disruption of the capillary endothelial barrier, resulting leakage of blood proteins into lung space and vice versa [27]. The appearance in plasma of proteins specific to the lung epithelium has been previously suggested to indicate loss of this barrier. Levels of lung surfactant proteins in serum have been reported to be elevated in response to pulmonary stress such as seen in COPD [28], pulmonary fibrosis [29] as well as in bacterial pneumonia [30]. Pulmonary surfactants are small phospholipoproteins that have been associated with diverse functions including, maintenance of alveolar function, innate immune defense especially in bacterial infections and are among the most abundant locally produced proteins in the BAL [31]. Our observation of a depletion of surfactant proteins from the murine BAL concomitant with their appearance in the serum is indicative of a ‘leakage’ of these lung proteins, presumably related to lung pathology caused by the influenza virus. To our knowledge this is the first report of BAL protein leakage into serum in influenza infections and raised the possibility of using this as a means of detecting lung injury in acute influenza infections. We tested this in a cohort of influenza and determined that significant amounts of at least two surfactants SPA and CC10 were detected in sera specifically during the acute febrile stage of infection. ROC curve analysis suggests that SPA had a significantly high predictive power in distinguishing influenza patients from asymptomatic controls. Levels of SPA also correlated with IL-6, which has been proposed as a marker for inflammatory damage in influenza. Although IL-6 levels were higher in our seasonal flu (H3N2) cohort, this was not statistically significant likely due to limited statistical power of this small cohort. Together, these observations suggest that specific lung proteins such as SPA have the potential to serve as tissue specific markers of lung damage in viral infections such as influenza. We also hypothesize that leakage of surfactants into the serum accompanies most influenza episodes, and may be proportional to the severity of lung damage. Use of more sensitive methods of detection of these proteins in the serum may facilitate earlier determination of lung damage especially in the context of severe influenza infections. Admittedly, clinical value of these markers in early detection of severe disease remains to be tested in patients with established severe lung pathology that were absent from the cohorts evaluated here. Establishing specificity of these markers to influenza, as well as statistical power of their performance will require follow up studies in larger and more diverse clinical cohorts. In conclusion, this study demonstrates that a subset of lung proteins may have tremendous potential as predictive biomarkers in severe influenza that will be improved by development of additional tools for high sensitivity measurement of these proteins in human sera.

## Supporting Information

Figure S1 **Temporal profile of BAL Cytokines in influenza infections.** Cytokines levels in BAL were measured as described in methods at indicated days following infection with PR8 influenza strains. The data is plotted as nanogram per ml and is shown as mean of data from 3 mice per group.
(TIF)

Figure S2 **Temporal serum cytokine profile in a human cohort of influenza patients.** Cytokine levels were measured in sera from two groups of human influenza cohort- one with influenza strain H3N2 (n = 26) and second infected with 2009 H1N1pdm09 virus strain (n = 30) as described in methods. In addition a separate cohort of asymptomatic healthy individuals is also shown as controls.
(TIF)

Table S1 **BAL Proteome flux in mouse model of infection- single peptide matches.**
(DOCX)
