# Supplementary figures and images for: Molecular Analysis of Serum and Bronchoalveolar Lavage in a Mouse Model of Influenza Reveals Markers of Disease Severity That Can Be Clinically Useful in Humans
Source: PLoS One. 2014 Feb 5;9(2):e86912. doi: 10.1371/journal.pone.0086912 (PMC3914809; doi:10.1371/journal.pone.0086912)

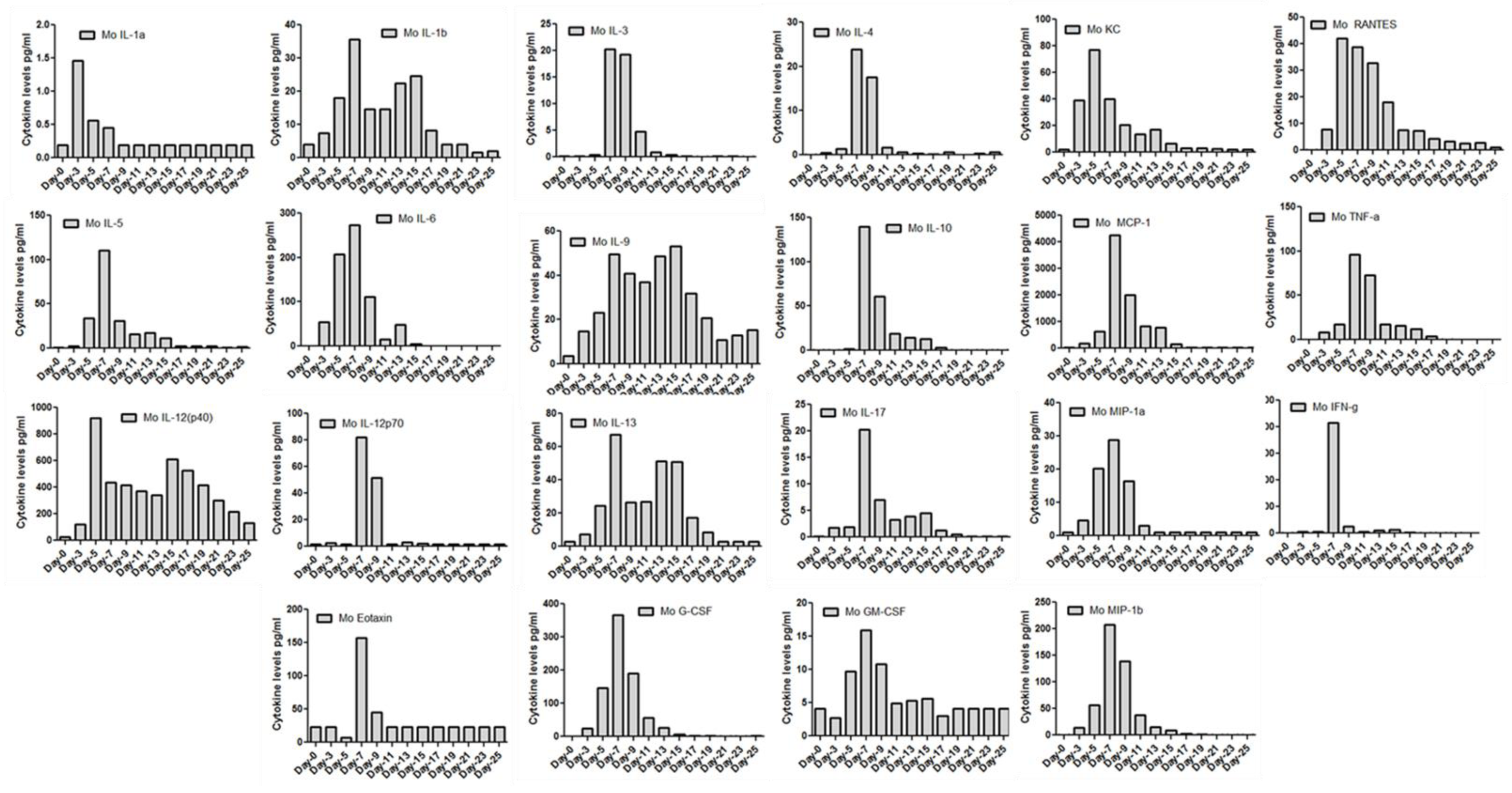

Supplement: Figure S1 — Temporal profile of BAL Cytokines in influenza infections. Cytokines levels in BAL were measured as described in methods at indicated days following infection with PR8 influenza strains. The data is plotted as nanogram per ml and is shown as mean of data from 3 mice per group. (TIF) [file pone.0086912.s001.tif]

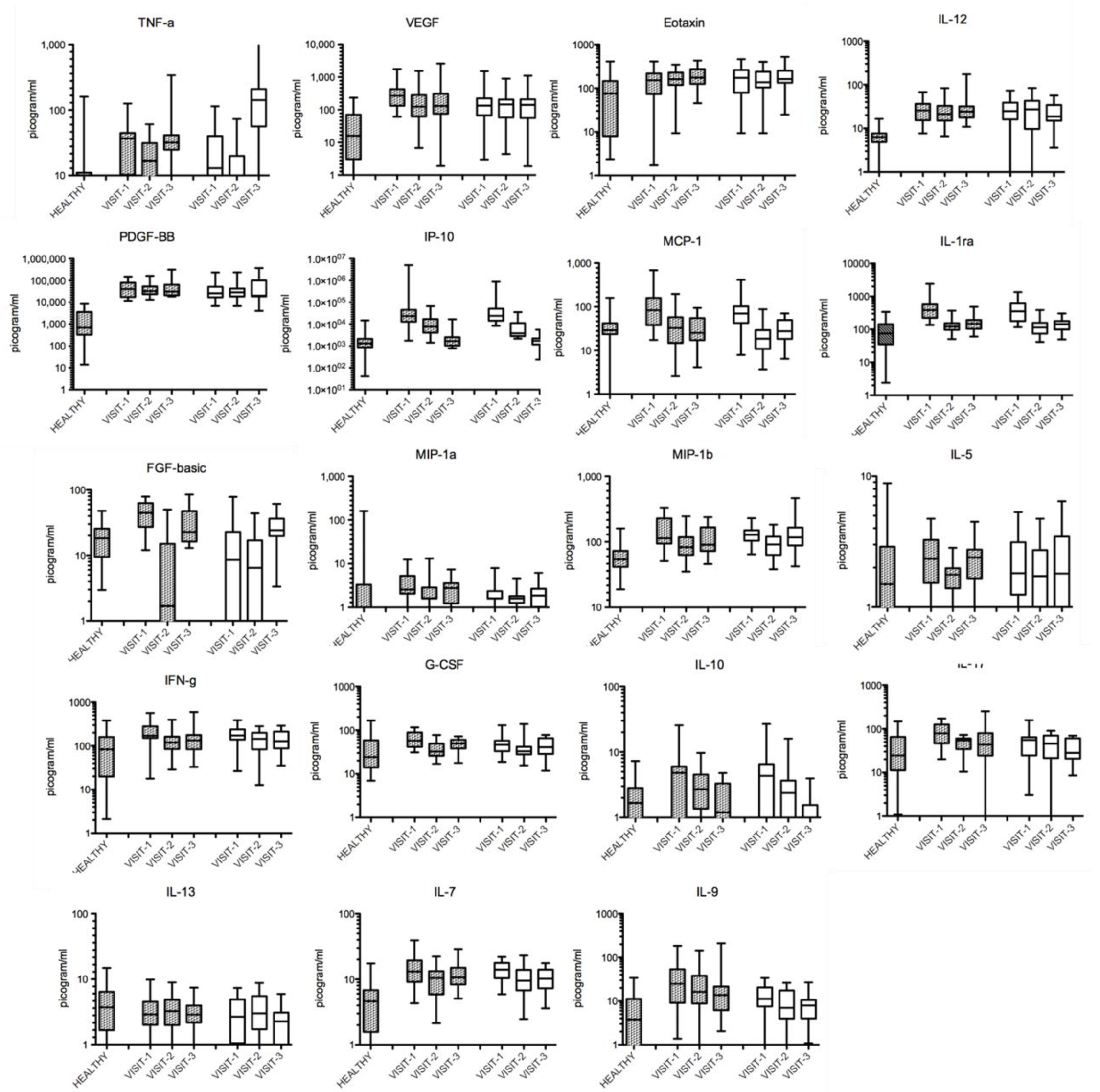

Supplement: Figure S2 — Temporal serum cytokine profile in a human cohort of influenza patients. Cytokine levels were measured in sera from two groups of human influenza cohort- one with influenza strain H3N2 (n = 26) and second infected with 2009 H1N1pdm09 virus strain (n = 30) as described in methods. In addition a separate cohort of asymptomatic healthy individuals is also shown as controls. (TIF) [file pone.0086912.s002.tif]
