# Supplementary material for: Molecular Analysis of Serum and Bronchoalveolar Lavage in a Mouse Model of Influenza Reveals Markers of Disease Severity That Can Be Clinically Useful in Humans
Source: PLoS One. 2014 Feb 5;9(2):e86912. doi: 10.1371/journal.pone.0086912 (PMC3914809; doi:10.1371/journal.pone.0086912)
Supplement: Table S1 — BAL Proteome flux in mouse model of infection- single peptide matches. (DOCX) [file pone.0086912.s003.docx]

| **BAL Protein** | **Swissprot Accession** | **No.of peptides** | **Fold Change over Day-0** | | |
| --- | --- | --- | --- | --- | --- |
|  |  |  | **Day-5** | **Day-14** | **Day-21** |
| Complement factor D | P03953 | 1 | 1.22 | 2.34 | 1.7 |
| Angiotensinogen | P11859 | 1 | 3.03 | 3.22 | 1.83 |
| **Glutathione S-transferase Mu 1** | **P10649** | **1** | **0.37** | **0.21** | **0.77** |
| **Protein Plunc** | **P97361** | **1** | **0.53** | **0.56** | **1.31** |
| Tubulin alpha-1B chain | P05213 | 1 | 1.46 | 1.75 | 1.8 |
| Osteopontin | P10923 | 1 | 1.05 | 2.66 | 1.52 |
| Transthyretin | P07309 | 1 | 1.7 | 3.53 | 2.04 |
| Heparin cofactor 2 | P49182 | 1 | 1.71 | 2.08 | 1.61 |
| Protein AMBP | Q07456 | 1 | 5.32 | 7.41 | 3.56 |
| **Peroxiredoxin-6** | **O08709** | **1** | **0.5** | **0.82** | **0.79** |
| **Superoxide dismutase [Cu-Zn]** | **P08228** | **1** | **0.45** | **0.44** | **0.61** |
| Moesin | Q61646 | 1 | 2.69 | 1.28 | 1 |
| Keratin, type II | O08677 | 1 | 0.79 | 0.47 | 0.49 |
| Aldehyde dehydrogenase, cytosolic 1 | P32261 | 1 | 0.82 | 0.69 | 1.29 |
| C-X-C motif chemokine 15 | P01942 | 1 | 0.79 | 0.97 | 0.93 |
| **Carbonyl reductase [NADPH] 2** | **Q01339** | **1** | **0.74** | **0.28** | **0.91** |
| Transforming growth factor-beta-induced protein ig-h3 | Q35744 | 1 | 3.34 | 4.08 | 2.96 |
| Inter-alpha-trypsin inhibitor heavy chain H1 | P28665 | 1 | 3.09 | 3.16 | 1.71 |
| Complement factor I | P13020 | 1 | 4.09 | 4.33 | 2.71 |
| 14-3-3 protein zeta/delta | P63260 | 1 | 1.42 | 1.19 | 1.05 |
| Alpha-1B-glycoprotein | P29699 | 1 | 7.12 | 1.95 | 2.06 |
| Glutathione peroxidase 3 | P02088 | 1 | 2.96 | 6.32 | 1 |
| Annexin A5 | Q06890 | 1 | 1.11 | 1.47 | 0.75 |
| Thioredoxin | P20918 | 1 | 1.26 | 1.42 | 1.45 |
| Intercellular adhesion molecule 1 | P50404 | 1 | 0.97 | 0.83 | 0.88 |
| Protein S100-A6 | Q06318 | 1 | 0.93 | 1.49 | 1.23 |
| Complement component C8 beta chain | Q61362 | 1 | 3.96 | 2.9 | 3.15 |
| **Alcohol dehydrogenase class 4 mu/sigma chain** | **P50405** | **1** | **0.31** | **0.66** | **1.7** |
| Extracellular superoxide dismutase [Cu-Zn] | Q89020 | 1 | 1.98 | 2.16 | 1.84 |
| Resistin-like alpha | Q8K0E8 | 1 | 3.61 | 5.38 | 5.02 |
| Cathepsin D | P04186 | 1 | 2.08 | 1.4 | 1.46 |
| Alpha-2-antiplasmin | P78417 | 1 | 0.78 | 3.14 | 1.03 |
| 14-3-3 protein gamma | P08226 | 1 | 1.64 | 1.57 | 0.71 |
| **Parvalbumin alpha** | **P08905** | **1** | **0.19** | **0.24** | **0.48** |
| Plasma protease C1 inhibitor | P01029 | 1 | 5.25 | 4.02 | 2.22 |
| Apolipoprotein D | Q63836 | 1 | 4.41 | 6.72 | 2.04 |
| Phospholipid transfer protein | P06909 | 1 | 2.37 | 2.32 | 1.72 |
| Heat shock protein HSP 90-beta | Q80X17 | 1 | 1.43 | 1.32 | 0.93 |
| Inter-alpha-trypsin inhibitor heavy chain H3 | Q9QXC1 | 1 | 7.59 | 11.87 | 3.71 |
| Fibrinogen gamma chain | Q61703 | 1 | 2.36 | 4.5 | 1.4 |
| Semaphorin-3F | P08071 | 1 | 5.95 | 0 | 0.46 |
| Histone H2A type 1-F | P35242 | 1 | 1.22 | 2.34 | 1.7 |
